# Toxicity and biochemical impact of methoxyfenozide/spinetoram mixture on susceptible and methoxyfenozide-selected strains of *Spodoptera littoralis* (Lepidoptera: Noctuidae)

**DOI:** 10.1038/s41598-022-10812-w

**Published:** 2022-04-28

**Authors:** Fatma S. Ahmed, Yasser S. Helmy, Walid S. Helmy

**Affiliations:** 1 Department of Economic Entomology and Pesticides, Faculty of Agriculture, Cairo University, Giza, 12613 Egypt; 2 Department of Biochemistry, Faculty of Agriculture, Cairo University, Giza, 12613 Egypt

**Keywords:** Biochemistry, Neuroscience, Chemistry

## Abstract

Methoxyfenozide (M) is one of the selective insecticides used in integrated pest management (IPM) programs for lepidopteran pests. However, recent studies reported a development of M-resistance, which prompted us to look for alternatives. Here, we investigate the potency of a mixture of M with spinetoram (Sp) on M-resistant insects. In the laboratory, a selection pressure with M has carried out on *Spodoptera littoralis* (Lepidoptera: Noctuidae) strains. A dipping technique was used to evaluate the toxicity of a sublethal concentration of M and Sp. on *S. littoralis* larvae, and the same concentrations were used to assess the toxic impact of their combination on susceptible (SUS) and M-selected (MS) strains. The toxicity of M/Sp mixtures was computed using a combination index equation, and a potentiation effect was observed in the two tested strains. Synergism tests revealed that piperonyl butoxide had considerable synergistic effects on M toxicity in the MS strain. The results revealed that the M/Sp mixture's negative effect on both monooxygenases and esterases is most likely the cause of its potentiation effect on the SUS and MS strains. It was concluded that M/Sp mixtures are effective against M-resistant *S. littoralis* strains, so these can be used in IPM programs.

## Introduction

In Africa, southern Europe, and the Middle East, the cotton leafworm *Spodoptera littoralis* (Boisduval) (Lepidoptera: Noctuidae) is a major polyphagous pest of numerous crops^1,2^. Its effects are not confined to cotton plants, as corn, clover, potato, sweet pepper, alfalfa, tomato, sweet potato, tobacco, castor, cabbage, peanuts, maize, soybeans, and eggplant are also attacked^3^. Since the 1950s, chemical management through conventional insecticides has been intensively used to combat this pest, resulting in resistance and environmental pollution. Consequently, researchers and manufacturers investigated alternative compounds that are effective against this pest and are safe for humans, environmentally friendly, and follow proper integrated pest management (IPM) protocols.

Thirty years ago, a novel class of insect growth regulators (IGRs) that act as 20-hydroxyecdysone agonists was discovered^4^. Methoxyfenozide (RH-2485), tebufenozide (RH-5992), and chromafenozide (ANS-118) are the main members of this IGR group that mimic the action of the steroid insect molting hormone 20-hydroxyecdysone (20E), which induces premature and incomplete molting, resulting in larval mortality^4^. These three chemicals are lepidopteran-specific compounds with negligible toxicity to mammals and non-target arthropods, such as insect pollinators and predators^5^.Methoxyfenozide (RH-2485) is the most recently commercially developed compound in this group, and it is the most potent analog to date against lepidopteran larvae^6^, including *S. littoralis*^7–9^, *S. exigua* (Hübner) (Lepidoptera: Noctuidae)^10^, and *Helicoverpa armigera* (Hübner) (Lepidoptera: Noctuidae)^11,12^, and *Plutella xylostella*^13^, dipteran pests *such as Culex pipiens*^14^, and *Musca domestica*^15^. In addition, methoxyfenozide is an environmentally friendly compound^16^ with less toxic effects on mammals, birds, fishes^17^, natural enemies such as the egg parasitoid of Helicoverpa species, Trichogramma nr. brassicae^18^, and beneficial insects such as Bumblebees *Bombus terrestris*^19^*.*

Unfortunately, in recent years, methoxyfenozide resistance has been reported in field populations of lepidopterous pests in several regions of the world, including the southern United States and Thailand^6^, Mexico^20^, Pakistan^21,22^, Spain^23^, China^21,24,25^, and Brazil^26^. Furthermore, an accelerated rate of resistance development was recorded when methoxyfenozide was selected in a laboratory^5,27,28^.

There are ways to avoid insecticide resistance in pests; one is to develop alternative classes of chemicals, while the other is to use insecticides that are likely to develop resistance in rotation or as mixtures of compounds with different modes of action^29^. These could be employed to manage resistant pest populations in the fields and postpone the development of insecticide resistance^29,30^.

Compared to when pesticides were used separately, pesticide combinations may result in a higher pest mortality^31^, less number of required applications^32^, and inhibited inception of resistance development in pest populations^33^. However, depending on the insect strain, physiology, and resistance mechanisms present in a population, these effects may differ^34^. Accordingly, determining the strength or weakness of an insecticide mixture and its detoxification mechanisms in the resistant strains deserves thoughtful attention.

Indeed, several investigations have shown that methoxyfenozide-containing mixtures have synergistic effects; however, the joint toxicity effect of methoxyfenozide and spinetoram mixtures on methoxyfenozide-resistant strains has not been yet documented. In this study, the effect of this combination on a methoxyfenozide-resistant strain of *S. littoralis* was investigated. In addition, the effects of this mixture on detoxification enzymes in susceptible and methoxyfenozide-selected strains were also explored.

## Materials and methods

### Susceptible strain

The susceptible strain of *S. littoralis* has established from egg batches collected from a cotton field at the agricultural research and experimental station, Faculty of Agriculture (University of Cairo), in the summer of 2018. Before the experiment began, this colony was reared in the laboratory for twelve generations without being exposed to insecticides. The strain was maintained at 26 °C ± 2 °C and 65% ± 5% relative humidity (RH) with a 16:8 h. light: dark photoperiod^35^. A 10% sugar solution was given to newly emerged moths, and they were allowed to lay their eggs on tissue paper. The collected eggs were maintained for hatching in other jars. Throughout the larval period, fresh castor oil plant leaves, *Ricinus communis* L., were supplied daily to the larvae. From this culture, second instar larvae were selected for bioassay tests. All experiments were performed in accordance with the relevant guidelines and regulations for use of plants. The castor plant was identified and authenticated by a Botanist at the Botany Department, Faculty of Agriculture, Cairo University, Egypt. Confirmation of the taxonomic identity of the plant was achieved by comparison with voucher specimens kept at the Egyptian Agriculture museum, and the use of documented literature^36^. The official permission of collecting castor plant leaves from greenhouses owned by Cairo university's Faculty of Agriculture for feeding insects and conducting research experiments was obtained from the vice dean for environmental affairs and community services sector.

### Selection with methoxyfenozide

The methoxyfenozide-selected strain was derived from a susceptible strain after 16 generations of treatment with 1–70 μg/mL of methoxyfenozide, which was specifically chosen for selection due to the current strain's accelerated rate of developing resistance to methoxyfenozide. Using leaf dipping bioassay technique^37^, the second instar larvae were exposed to the pesticide at a concentration comparable to the LC_50_ of the baseline set for the laboratory colony in the first round of selection. Surviving larvae were transferred to untreated castor leaves and reared in the laboratory under the conditions specified above after 24-h exposure. During selection cycles, the mortality ranged from 10 to 90%. Based on the results of the previous generation's bioassays, the methoxyfenozide concentration utilized to select each successive generation was LC_50_. Depending on availability, the number of second instar larvae used for each generation varied (1000–2000).

### Chemicals

Spinetoram (Radiant^®^ 12%SC), a spinosyn, and methoxyfenozide (Runner^®^ 24%SC), a diacylhydrazine, were both provided by Dow Agro Sciences. The following chemicals were purchased from Sigma-Aldrich (Sigma-Aldrich, St. Louis, MO): Piperonyl butoxide (PBO), diethyl maleate (DEM), triphenyl phosphate (TPP), monopotassium phosphate (KH_2_PO_4_), dipotassium phosphate (K_2_HPO_4_), ethylenediaminetetraacetic acid (EDTA), α-naphthyl acetate, fast blue B salt, 1-chloro-2,4-dinitrobenzene (CDNB), L-glutathione reduced (GSH), 7-ethoxycoumarin (7-EC), and β-nicotinamide adenine dinucleotide phosphate (reduced β-NADPH).

### Bioassay

In three independent experiments, a leaf dipping bioassay technique^37^ was used. Lethal concentration (LC) values were determined using a range of five to seven serial concentrations of each insecticide (diluted with tap water). Castor plant leaves were dipped in each prepared concentration for 20 s before drying at room temperature (29 ± 2 °C) for 1 h. One hundred of the second instar larvae of the susceptible strain were placed in glass jars covered with a clean muslin cloth and divided into five replicates (20 larvae/replicate). The larvae were starved for 4 h before feeding and were allowed to feed on the treated leaves for 24 h. Any living larvae were transferred to clean jars with new untreated castor leaves after 24 h. Abbott's formula was used to correct the mortality percentages after 96 h^38^. The toxicity index, which is the ratio between the LC_50_ of the most toxic insecticide and the LC_50_ of our tested insecticide multiplied by 100, was calculated^39^. For the analysis of synergistic effects, PBO, DEM, and TPP were dissolved in acetone. Toxicity was first determined using a range of synergist concentrations to find a suitable concentration that did not affect larval mortality. Concentrations up to 100 mgL^−1^ of these synergists had no effect on larval mortality (*P* > 0*.*05). After 96 h, larvae mortality was recorded. The synergism ratio (SR) was calculated by dividing the LC_50_ of insecticide alone by the LC_50_ of the insecticide with a synergist.

### Binary mixtures

The calculated LC_25_ of spinetoram was prepared twice: once with the LC_25_ of methoxyfenozide on the susceptible strain and once with the LC_25_ of methoxyfenozide on the methoxyfenozide-selected strain. Each binary mixture was diluted five to seven times in bioassays, with a serial dilution factor of two. Using the same bioassay method described previously, the second instar larvae of susceptible and methoxyfenozide-selected *S. littoralis* strains were subjected to each dilution in three replicate samples. The combination index (CI)^40^ was adopted to quantify the potentiation (CI < 1), additive (CI = 1), or antagonistic (CI > 1) effects. Based on the bioassay results, the CI values at 10, 50, and 90 percent mortality rates were calculated using the CompuSyn software (www.combosyn.com).

$$n\left( {CI} \right)X = \mathop \sum \limits_{j = 1}^{n} \frac{\left( D \right)j}{{\left( {Dx} \right)j}} = \mathop \sum \limits_{j = 1}^{n} \frac{{\left( {Dx} \right)1 - n\left\{ {\frac{\left[ D \right]j}{{\mathop \sum \nolimits_{1}^{n} \left[ D \right]}}} \right\}}}{{\left( {Dx} \right)j\left\{ {\frac{{\left( {fax} \right)j}}{{\left[ {1 - \left( {fax} \right)j} \right]}}} \right\}1/mj}}$$

where *n*(*CI*)*X* is the combination index for *n* insecticides at *x*% mortality rate, (*Dx*)1 − *n* is the sum of the concentrations of *n* insecticides causing *x*% mortality in insecticide combination, [*D*] *j*/Σ1 *n* [*D*] is the proportion of concentration of each of *n* insecticides causing *x*% mortality in insecticide combination, and (*Dx*)*j*{(*fax*)*j*/ [1 − (*fax*)*j*]1/*mj*} is the concentration of each insecticide causing *x*% mortality rate.

### Enzyme assays

#### Esterase assay

After 96 h, twenty-live larvae of the treatment or control groups were weighed, rinsed with distilled water, and homogenized in 40 mM potassium phosphate buffer containing 1 mM EDTA at pH 7. Then, the homogenates were centrifuged at 12,000 rpm for 10 min using Sigma-3K30 Centrifuge. Co.UK. The supernatants were transferred into a clean Eppendorf. The α-esterase activity in total units (μ moles/mL/min) was determined according to the^41^ method with some modification. Briefly, 50 µL of α-naphthyl acetate solution (30 mM α-NA in acetone) and 50 µL of larval homogenate supernatant were incubated for 15 min at 25 °C. Then, 50 µL of staining solution (1% fast blue B salt in ethanol [w/v] and 5% sodium dodecyl sulfate [SDS] in distilled water w/v in 2:5 ratio) was added and the total volume was made up to 1 mL with PPB ( 40 mM, PH 7). The enzyme activity was read at 600 nm as an endpoint (Spectrophotometer UV–VIS, Shimadzu UV-1201), and the absorbance levels were compared with a standard curve of absorbance for known concentrations of α-naphthol (50 mM methanolic stock solution). Three replicates at least for each treatment and control were used. The α-esterase-specific activities were reported as [µmoles of α-naphthol formed min^–1^ mg^–1^ protein].

#### Glutathione-S-transferase assay

After 96 h, twenty larvae of each treatment and control were weighed, rinsed with distilled water, and homogenized in 100 mM potassium phosphate buffer containing 1 mM EDTA at pH 6.5. Then, the homogenates were centrifuged at 10,000 rpm for 10 min. The supernatants were transferred into a clean Eppendorf^42^ method was used to determine the Glutathione-S-transferase (GST) activity with some modifications. Briefly, 3 ml of the reaction mixture was made from 150 µL of 50 mM reduced L-glutathione (GSH), 50 µL of 50 mM CDNB, and 30 µL of the sample supernatant. The absorbance increment at 340 nm was recorded at a 1-min interval against a blank for 5 min. An extinction coefficient of 9.6 mM/cm was used to calculate the amount of CDNB conjugated. Three replicates were used to determine the GST activity for each treatment and control. The GST-specific activities were expressed as [nmols min^–1^ mg^–1^ protein].

#### Fluorometric monooxygenase (MO) determination

MO activity was determined using the^43^ methodology and detailed by Van Pottelberge et al*.*^44^ with some modifications. Moving larvae were collected after 96 h of methoxyfenozide treatment (G16) or not (untreated), and five midguts were dissected from each replicate. The midguts were rinsed in 900 μL of ice-cold 0.1 M phosphate buffer (pH 7.6) containing 1 mM EDTA. The midguts were homogenized and centrifuged at 4 °C for 20 min at 10,000 rpm. The supernatants were collected for testing the cytochrome P450 MO activity using 7-EC as a substrate. Exactly 50 μL of homogenate supernatant was mixed with 50 μl of the reaction mixture (0.1 M potassium phosphate buffer [pH 7.2] containing 1 mM EDTA, 0.4 mM 7-EC in methanol, and 1 mM NADPH) in each well of a FLUOstar^®^ Omega multi-mode microplate reader (BMG Labtech Ltd, Aylesbury, United Kingdom). The plate was incubated for 30 min at 30 °C in the dark while being gently shaken. To oxidize NADPH, 100 mM of GSSG in distilled water and 0.1 unit/μL of glutathione reductase were added to each well at 37 °C for 15 min. The reaction was stopped with 100 μL of 50% (v/v) acetonitrile in 50 mM Tris/ HCl buffer (pH 10). The fluorescence of 7-EC was measured at 460 nm while exciting it at 360 nm. The MO activity (7-EC-O-deethylation) was determined based on the 7-EC standard curve^45^ to convert the initial velocity to activity. The MO activity was expressed as pmols of 7-hydroxycoumarin formed/min/mg protein.

#### Protein assay

Bradford's method^46^ was used to estimate the total protein content using Coomassie brilliant blue dye and bovine serum albumin as a standard. For each larval homogenate, three replicates of 20 μL were tested. After 5 min, the OD at 595 nm was measured against blanks and was converted to a protein concentration (mg/mL) using the standard curve of absorbance of known concentrations of bovine serum albumin.

### Statistical analysis

The mortality percentages were corrected when needed and were subjected to probit analysis^47^ using a Log Dose Probit line^®^ program (http://www.ehabsoft.com/ldpline) to estimate the LC values and their corresponding 95% fiducial limits (FL). The fiducial limits indicate the required lethal dose to achieve 50 or 90 percent mortality in the study population within the lower and upper limits with 95 percent confidence. Dose–effect curve parameters and CI values were calculated with CompuSyn software^48^. The results of the enzyme assays are presented as mean ± SEM and were analyzed by one-way analysis of variance followed by Tukey’s post-hoc test (*P* < 0.01) using an SPSS software (version 15.0, SPSS Inc., Chicago, IL, U.S.A).

## Results

### Methoxyfenozide resistance selection

**Figure 1:**
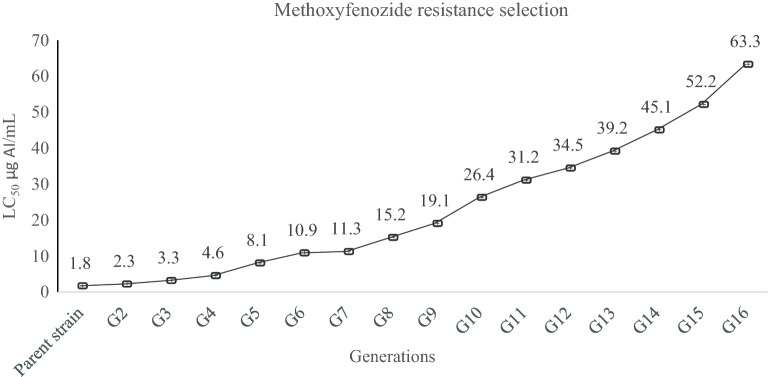
The LC_50_ (μg/mL) values of methoxyfenozide during selection with increasing concentrations towards *S. littoralis* second instar larvae over 16 generations.

The insects rapidly developed resistance to methoxyfenozide when unceasingly selected with increasing concentrations under laboratory conditions. The LC_50_ value was increased to 63.35 mg L^−1^ after 16 generations of selection, compared to 1.748 mg L^−1^ for the beginning susceptible colony (Fig. 1). These data indicate that the selected strain developed a 36.2-fold increase in resistance toward methoxyfenozide (M) during the selection processes.

### Synergistic effect

Table 1 shows the synergistic effects of PBO, DEM, and TPP with methoxyfenozide against susceptible (SUS) and methoxyfenozide selected (MS) strains of *Spodoptera littoralis*. The synergists tested did not affect the toxicity of methoxyfenozide in the SUS strain; however, in the MS strain, PBO produced a 3.33-fold synergism. TPP synergy was not observed in either strain. DEM reduced methoxyfenozide toxicity in the SUS strain, but it increased it in the MS strain.

**Table 1:** Synergism of PBO, DEM, and TPP on methoxyfenozide in the 2nd instar larvae of susceptible (SUS.) and methoxyfenozide selected (MS) strains of *Spodoptera littoralis* after 96 h. post-treatment.

Strain	Treatments	LC_50_ (µg AI mL^-1^) (95% FL)	Slope (SE)	X^2^ (df)	*g* value	SR*
SUS	M	1.748 (1.336–2.141)	1.75(0.22)	3.79 (3)	0.09	–
	M. + PBO	1.489 (1.233–1.733)	2.58(0.25)	3.99 (3)	0.03	1.17
	M. + DEM	2.195 (1.883–2.504)	2.65(0.24)	4.75 (3)	0.03	0.79
	M. + TPP	1.885 (1.574–2.191)	2.39(0.21)	4.21 (3)	0.04	0.93
MS. (G16)	M	63.31 (53.32–76.22)	1.57(0.17)	7.13 (3)	0.04	–
	M. + PBO	18.99 (16.17–21.92)	2.67(0.36)	2.46(1)	0.03	3.33
	M. + DEM	55.65 (47.54–65.23)	1.77(0.18)	7.59(4)	0.04	1.14
	M. + TPP	64.80 (55.79–74.17)	2.19(0.30)	5.20(3)	0.07	0.98

*M* Methoxyfenozide, *PBO* Piperonyl Butoxide, *DEM* Diethyl Maleate, and *TPP* Triphenyl Phosphate. LC_50_ is the concentration, in μg (microgram) of methoxyfenozide per mL (milliliter) of water, that is required to kill 50% of the tested population. *X*^2^ chi-square, and *df* Degree of freedom. *g value* goodness of fit, and *SR (synergism ratio) = LC_50_ without synergist/LC_50_ with synergist.

### Toxicity of the tested insecticides alone on susceptible and resistant strains

Table 2 lists the results of the toxicity test for the tested insecticides with their 95% FL. The toxicity of spinetoram (Sp) was significantly higher (*P* < 0*.*01; non-overlapping of 95% FL) than that of methoxyfenozide toward the susceptible strain of *S. littoralis*. The 96-h LC_50_ values of methoxyfenozide and spinetoram tested against the second instar larvae of the laboratory strain were 1.748 and 0.038 µg AI ml^−1^, respectively, while the LC_25_ values used in the mixture preparation were 0.684 and 0.009 µg AI ml^−1^, respectively.

**Table 2:** Toxicity (LC values) of methoxyfenozide and spinetoram individually to the 2nd instar larvae of a susceptible of *Spodoptera littoralis* after 96 h. post-treatment.

Insecticides	No	LC_25_ (µg AI mL^-1^) (95% FL)	LC_50_ (µg AI mL^-1^) (95% FL)	Slope (SE)	X^2^ (df)	*g* value	TI* (%) at LC_50_
	**Susceptible strain**						
Methoxyfenozide	100	0.684 (0.420–0.951)	1.748 (1.336–2.141)	1.75(0.22)	3.79(3)	0.09	2.17
spinetoram	100	0.009 (0.003–0.016)	0.038 (0.026–0.051)	1.1 (0.18)	0.05(3)	0.1	100

*No*. number of larvae exposed to the insecticide, LC_25,_ and LC_50_ are concentrations of each insecticide, in μg (microgram) of insecticide per mL (milliliter) of water, that is required to kill 25 or 50% of the tested population, respectively. *X*^2^ chi-square, and *df* Degree of freedom. *g value* goodness of fit, and *Toxicity index^39^ = LC_50_ of the most efficient compound/LC_50_ of the other compound × 100.

In M-resistant strain, the selection (G0 to G16) increased the resistance ratio (RR) for M by 36-fold, with an LC_50_ value of 63.31 µg mL^−1^. The LC_50_ value of spinetoram was increased to 0.124 µg AI mL^−1^, with a three-fold increase in RR. Methoxyfenozide and spinetoram had 96-h LC_25_ values of 23.65 and 0.031 µg AI ml^−1^ against the second instar larvae of the resistant strain, respectively (Table 3).

**Table 3:** Toxicity (LC values) and resistance ratio of methoxyfenozide and spinetoram individually to the 2nd instar larvae of a methoxyfenozide selected strain of *Spodoptera littoralis* after 96 h. post-treatment.

Insecticides	No	LC_25_ (µg AI mL^-1^) (95% FL)	LC_50_ (µg AI mL^-1^) (95% FL)	Slope (SE)	X^2^ (df)	*g* value	RR_50_*
		**Methoxyfenozide- selected strain**					
Methoxyfenozide	100	23.65 (17.53–29.40)	63.31 (53.32–76.22)	1.57(0.17)	7.13(3)	0.04	36
Spinetoram	100	0.031 (0.016–0.047)	0.124 (0.089–0.164)	1.11(0.13)	5.54(2)	0.05	3

*No*. number of larvae exposed to the insecticide, LC_25_, and LC_50_ are concentrations of each insecticide, in μg (microgram) of insecticide per mL (milliliter) of water, that is required to kill 25 or 50% of the tested population, respectively. *X*^2^ chi-square, and *df* Degree of freedom. *g value* goodness of fit, and *RR_50_ (Resistance Ratio)^62^ = LC_50_ of tested generation/ LC_50_ of parent strain.

### Toxicity of spinetoram/methoxyfenozide mixture on the laboratory and methoxyfenozide-resistant strains

The LC_50_, slope (m), and linear correlation coefficient (r) of M/Sp mixture on the SUS and MS strains of *S. littoralis* and the average CI values for three representative effect levels (LC_10_, LC_50_, and LC_90_) are shown in Table 4. In the laboratory and MS strains, the LC_50_ of the M/Sp combination increased from 0.046 to 62.32 µg AI ml^−1^, respectively. Despite this, the M/Sp combination demonstrated an extremely strong potentiation in both strains (Table 4).

**Table 4:** Dose–effect relationship parameters and mean combination index (CI) values of the methoxyfenozide (M.)/spinetoram (Sp.) mixtures on laboratory (Susceptible) and resistant strains of *S. littoralis.*

Mixtures		Dose–effect parameters			CI values					
	Strains	LC_50_	m	r	LC_10_	Graded symbols	LC_50_	Graded symbol	LC_90_	Graded symbols
M. + SP	SUS	0.046	1.29	0.98	0.10	++++	0.05	+++++	0.03	+++++
M. + SP	MS	62.32	1.16	0.96	0.15	++++	0.06	+++++	0.02	+++++

The parameter m is the slope of the median-effect plot (which signifies the shape of dose–effect curve), and r is the linear correlation coefficient (which signifies the conformity of data to the mass-action law. LC 50 and m are used for calculating CI values, CI < 1, CI = 1, and CI > 1 indicate synergism, additive effect, and antagonism, respectively.LC_10_ LC_25_, and LC_50_ are the concentrations required to reach a response mortality of 10, 25, and 50%, respectively. Graded symbols (++ + ++) very strong potentiation, (++ ++) strong potentiation, (+++) potentiation, (++) moderate potentiation, (+) slight potentiation^40,48^.

### Detoxification enzymes

#### Carboxylesterase activity

**Figure 2:**
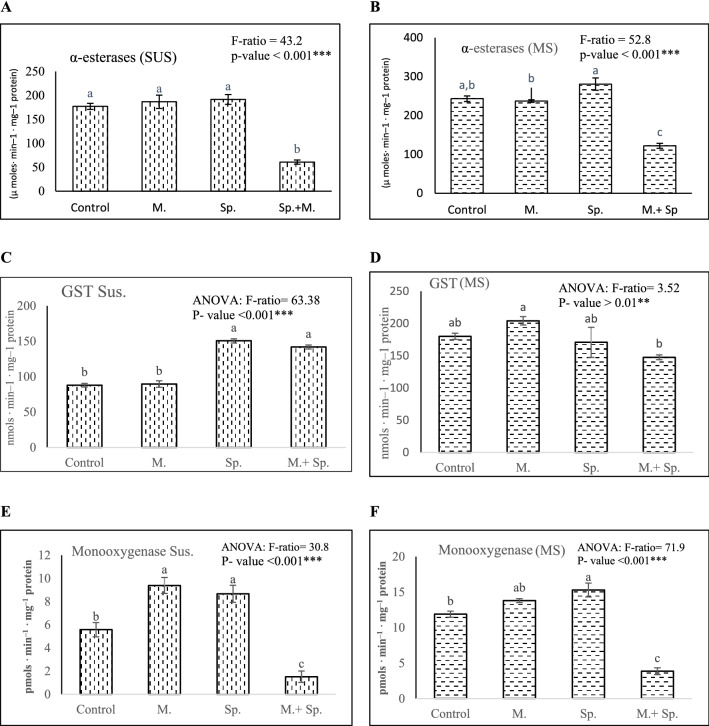
Enzymes specific activity 96 h. post-treatments with LC_25_ of Spinetoram (Sp), LC_25_ of Methoxyfenozide (M), or LC_25_: LC_25_ values of M + Sp on the susceptible (SUS) and resistance (MS) strains of *Spodoptera littoralis*, respectively. (**A**), and (**B**): α-esterase activity, (**C**) and (**D**): GST activity, and (**E**) and (**F**): Monooxygenase activity. Values represent mean ± standard error. Means followed by different letters are significantly different according to Tukey’s multiple range comparison (*P* < 0.01). F-ratio and *p*-values are calculated with ANOVA analysis.

When compared to the control, the sublethal concentration (LC_25_) of methoxyfenozide and spinetoram did not affect the alpha-esterase-specific activity (µ moles min^–1^ mg^–1^ protein) in either the laboratory (F = 43.2, *P* < 0.001) or resistant (F = 52.8, *P* < 0.001) strains, but the M/Sp mixture showed statistically significant inhibition in their activity (Fig. 2A and B).

#### Glutathione-S-transferase activity

As shown in Fig. 2C, spinetoram alone and in combination with methoxyfenozide significantly increased the GST-specific activity (nmol min^–1^ mg^–1^ protein) in the laboratory strain (F = 63.38, *P* < 0.001), but methoxyfenozide alone had no effect when compared to the control. The GST-specific activity in the resistant strain did not differ significantly between the treatments (F = 3.25, *P* > 0.01) (Fig. 2D).

#### Monooxygenase activity

In the laboratory strain (SUS), the sublethal concentration (LC_25_) of Sp or methoxyfenozide individually significantly increased the MO activity (pmols min^–1^ mg^–1^ protein) (F = 30.8, *P* < 0.001), but the M/Sp mixture showed statistically significant inhibition in its activity when compared to the control (Fig. 2E).

However, in the MS strain, the MO activity did not change statistically when treated with the sublethal concentration of methoxyfenozide compared to the control, but the M/Sp mixture showed statistically significant inhibition in its activity (F = 71.9, *P* < 0.001) (Fig. 2F).

## Discussion

In this study, the methoxyfenozide (M) resistance laboratory-selected *S. littoralis* showed reduced susceptibility to methoxyfenozide after 16 continuous generations. The LC_50_ increased from 1.74 μg/mL in the parent strain to 63.31 μg/mL (Fig. 1). This result agrees with^27^, as they successfully selected a field-collected colony of *S. exigua* for methoxyfenozide resistance after only seven generations. In addition, Moulton et al.^6^ reported a 120-fold increase in methoxyfenozide resistance in a field population of *S. exigua* after a few generations of selection.

*S. littoralis* is a swarming, polyphagous, foliage-feeding insect found worldwide. This insect is one of the most frequent cotton pests, wreaking havoc on various crops^49^. One of the most important problems in this pest is its resistance to almost all chemical groups used against it^50^. Consequently, it has sparked a lot of interest in finding ways to avoid or overcome this problem. For example, insecticide mixtures may present intriguing possibilities for pest management, particularly if potentiation interactions among insecticides occur^51^. As stated by Ahmad^34^, mixing pesticides with different modes of action may delay the development of resistance within pest populations. This is because the resistance mechanisms required for each pesticide in the mixture may not be widely distributed or exist in insect populations^52^.

This study assessed the insecticidal effects of the LC_25_ value of methoxyfenozide (Ecdyson agonist), and the same value of spinetoram (Sp) (activator for the nicotinic acetylcholine receptors) individually and in combination against SUS and MS strains of *S. littoralis*. The adoption of these LC_25_ values of the M/Sp mixture is based on preliminary experiments. The use of concentrations greater than the LC_25_ values of both compounds in the form of a mixture resulted in a mortality of nearly all treated insects after 96 h of treatment, making it impossible to calculate the LC values or to conduct enzyme assays, which reflect the mixture's potency on the tested strains.

Depending on the LC_50_ values of both compounds, they are considered highly toxic to the SUS strain of *S. littoralis* (Table 2). Furthermore, in the MS strain, there was no cross-resistance between Sp and methoxyfenozide, as the RR for Sp after 16 generations of selection pressure with M was 3 (Table 3). This finding agrees with^26^, who reported a negative cross-resistance between methoxyfenozide and spinetoram in the MS strain of *Tuta absoluta* (Meyrick) (Lepidoptera: Gelechiidae). They suggested using spinetoram to mitigate the methoxyfenozide resistance in the field.

In insects, the detoxification process involves adding functional groups to lipophilic xenobiotics, primarily through oxidation–reduction and/or hydrolysis reactions carried out by phase I enzymes like cytochromes P450s and carboxylesterases (CaEs). Then, phase II enzymes such as GSTs conjugate phase I metabolites into small hydrophilic molecules^53^. These detoxification enzymes, MO, CaEs, and GST, have been reported to gain the most significant role in insect resistance to either synthetic or non-synthetic insecticides^52,54^. Globally, the resistance is mostly associated with increased levels of these detoxifying enzymes in insecticide-resistant populations^55^.

In this study, using the LC_25_ values of methoxyfenozide and spinetoram individually did not statistically change the esterase-specific activity compared to the control group in the SUS and MS strains (Fig. 2A and B), indicating that esterases are insensitive to these compounds. In many insect species, increased esterase activity is a major mechanism of insecticide insensitivity or resistance^56^.

In contrast, the LC_25_ values of methoxyfenozide and spinetoram individually elevated the activity of MO enzymes in the SUS and MS strains (Fig. 2E and F); indicating that these enzymes may have a role in the degradation of these two compounds. methoxyfenozide showed considerable synergism with PBO in the MS strain, which agrees with^28^, indicating that MO was involved in resistance. Metabolic enzymes have been linked to methoxyfenozide resistance in cotton leafworm *S. littoralis*^5^ and *H. armigera*^57^. Moreover, the involvement of MO in the mechanism of spinosad resistance was reported in *S. exigua*^58,59^. Sial et al*.*^60^ also recorded the same result when Sp was used against *Choristoneura rosaceana* (Harris) (Lepidoptera: Tortricidae). These results were expected as spinosad and spinetoram are both spinosyns.

One of the intriguing findings in this study is the significant decrease in MO activity after 96 h of treatment with a mixture of sublethal concentrations of methoxyfenozide and spinetoram in both SUS and MS strains (Fig. 2E and F). This finding suggests that the potency of this mixture may be attributed to the ability of both compounds to disrupt the insect's detoxification metabolic pathway of these compounds. This conclusion is supported by the significant decrease in the activity of esterase enzymes after the treatment with the same mixture in the SUS and MS strains, while the activity of esterase enzymes activity did not change when each compound was used individually.

However, some resistance mechanisms in *S. littoralis*, such as increased MO detoxification^61^, may nullify the benefits of pesticide combinations. Moreover, mixtures may also give way to new resistances, which may expand to other chemical classes and become challenging to handle^34^. Fortunately, this study found no evidence of M-Sp cross-resistance. This finding, together with the resistant strain's high level of sensitivity to this mixture, implies that using this mixture against *S. littoralis* is useful in avoiding the rapid development of M resistance.

This study highlighted the importance of testing insecticide mixtures on resistant pest strains. The mixture's success on susceptible strains does not necessarily imply its success on resistant strains, which are typically found in the fields. Additionally, one significant benefit of using the mixture suggested in this study is that both its components are very safe for mammals and non-target organisms, and they do not pollute the environment. It is also expected that using low concentrations of both compounds to manage lepidopteran pests associated with cotton will have no negative effects on biological systems or the environment. However, further research on this mixture is required to test its chronic toxicity to mammals. Furthermore, the GST activity was measured using the conjugation of CDNB, which demonstrated no significant differences between any of the treatments and the unselected colony in the MS strain (Fig. 2D). However, more research should be done using both CDNB and 1,2-dichloro-4-nitrobenzene to see if GSTs are involved in the detoxification process.

## Supplementary Information

Supplementary Information.

## Data Availability

All data generated or analyzed during this study are included in this published article and its supplementary information file.
