# Supplementary material for: Toxicity and biochemical impact of methoxyfenozide/spinetoram mixture on susceptible and methoxyfenozide-selected strains of Spodoptera littoralis (Lepidoptera: Noctuidae)
Source: Sci Rep. 2022 Apr 28;12:6974. doi: 10.1038/s41598-022-10812-w (PMC9050723; doi:10.1038/s41598-022-10812-w)
Supplement: Supplementary file 1 — Supplementary Information. [file 41598_2022_10812_MOESM1_ESM.pdf]

Toxicity and biochemical impact of methoxyfenozide/spinetoram mixture on susceptible and methoxyfenozide-selected strains of *Spodoptera littoralis* (Lepidoptera: Noctuidae)

Fatma S. Ahmed[a*]; Yasser S. Helmy[b]; Walid S. Helmy[a]

[a]Department of Economic Entomology and Pesticides, Faculty of Agriculture, Cairo University, 12613 Giza, Egypt
[b]Department of Biochemistry, Faculty of Agriculture, Cairo University, 12613 Giza, Egypt
***Author for correspondence, Email:** fatma.sherif@cu.edu.eg

Table. The slope, LC_{50} (µg/mL), and total number of *Spodoptera littoralis* 2nd instar larvae exposed to increasing concentrations of methoxyfenozide during selection over 16 generations.

Generations	Slope	LC_{50} µg *a.i.*/mL	95% FL* (Lower- Upper)	X^2	g value	RR_{50}**	Total number of the 2nd instar larvae exposed to LC_{50} of methoxyfenozide selection.
**Parent**	1.66±0.18	1.8	1.33-2.14	3.79	0.05	-	
**G2**	1.84±0.15	2.3	1.93-2.64	1.03	0.03	1.3	1200
**G3**	1.80±0.17	3.3	2.75-3.80	6.86	0.03	1.8	1400
**G4**	2.02±0.18	4.6	4.02-5.33	7.74	0.03	2.6	2000
**G5**	4.61±0.41	8.1	7.64-8.65	1.40	0.03	4.5	1300
**G6**	2.50±0.22	10.9	9.89-12.1	6.22	0.03	6.1	1000
**G7**	2.29±0.26	11.3	9.99-12.7	6.36	0.05	6.3	1600
**G8**	2.95±0.26	15.2	13.8-16.7	6.01	0.03	8.5	1300
**G9**	0.26±0.29	19.1	16.9-21.2	0.69	0.05	10.6	1000
**G10**	3.65±0.43	26.4	23.9-28.5	3.37	0.05	14.6	1300
**G11**	4.15±0.39	31.2	28.6-33.5	1.53	0.03	17.3	1500
**G12**	2.82±0.30	34.5	30.5-38.2	3.02	0.04	19.2	1100
**G13**	3.33±0.28	39.2	35.8-42.7	5.37	0.03	21.8	1000
**G14**	2.78±0.25	45.1	40.9-49.4	1.38	0.03	25.1	1000
**G15**	1.92±0.26	52.2	43.4-60.2	7.70	0.07	29.0	1200
**G16**	1.58±0.17	63.3	53.3-76.2	7.13	0.05	35.2	1800

* Fiducial limits at 90% probability level
** RR_{50} (Resistance Ratio) = LC_{50} of tested generation/ LC_{50} of parent strain.

Tables. Absorbance of α- esterases, GST and Monooxygenase samples 96 hrs. post-treatments with LC_{25} of Spinetoram (Sp), LC_{25} of Methoxyfenozide (M), or LC_{25}: LC_{25} values of M+ Sp on the susceptible (SUS) and resistance (MS) strains of *Spodoptera littoralis*, respectively.

Treatments	α- esterases on SUS. Strain			α- esterases on MS. Strain		
	abs. R1	abs. R2	abs. R3	abs. R1	abs. R2	abs. R3
Control	0.972	0.992	0.893	1.231	1.460	1.312
spinetoram (Sp.)	0.695	0.786	0.679	1.230	1.035	1.012
Methoxyfenozide (M.)	0.495	0.408	0.448	0.768	0.925	1.120
SP+M	0.236	0.258	0.271	0.358	0.321	0.359

Treatments	GST on SUS. Strain						GST on MS. Strain					
	R1		R2		R3		R1		R2		R3	
	initial Abs.	final Abs.	initial Abs.	final Abs.	initial Abs.	final Abs.	initial Abs.	final Abs.	initial Abs.	final Abs.	initial Abs.	final Abs.
Control	0.882	0.926	0.685	0.726	0.685	0.725	0.647	0.734	0.811	0.911	0.901	0.985
Spinetoram (Sp.)	0.895	0.985	1.245	1.330	1.335	1.425	0.801	0.992	0.568	0.734	0.798	0.968
Methoxyfenozide (M.)	0.900	0.992	0.647	0.734	0.875	0.968	0.842	0.985	1.214	1.33	1.258	1.425
SP+M	0.655	0.708	0.654	0.753	0.642	0.739	0.656	0.708	0.653	0.753	0.725	0.831

Treatment	Monooxygenase on SUS. Strain			Monooxygenase on MS. Strain		
	abs. R1	abs. R2	abs. R3	abs. R1	abs. R2	abs. R3
Control	1.003	0.956	0.958	1.124	1.111	1.147
spinetoram (Sp.)	1.068	1.036	1.095	1.201	1.211	1.225
Methoxyfenozide (M.)	1.036	1.025	1.085	1.192	1.201	1.125
SP+M	0.896	0.856	0.865	0.923	0.953	0.914
